# Performance evaluation of the new Dymind automated hematology analyzer

**DOI:** 10.1016/j.plabm.2025.e00507

**Published:** 2025-09-22

**Authors:** Tipparat Penglong, Wanicha Tepakhan, Nasra Tehyoh, Yanisa Na Songkhla, Chakkrit Songnak, Kanitta Srinoun

**Affiliations:** a Department of Pathology, Faculty of Medicine, Prince of Songkla University, Hat Yai, Songkhla, 90110, Thailand; b Centre for Research and Development of Medical Diagnostic Laboratories, Faculty of Associated Medical Sciences, Khon Kaen University, Khon Kaen, 40002, Thailand; c Faculty of Medical Technology, Prince of Songkla University, Hat Yai, Songkhla, 90110, Thailand

**Keywords:** Complete blood count, Automated hematology analyzer, Method comparison, Dymind series, Between-run precision

## Abstract

**Introduction:**

The Dymind automated hematology analyzer includes the five-part analyzers DF55 and DH76 and the six-part analyzer DH615, which features reticulocyte parameters and an artificial intelligence-driven analysis technology. This study evaluated the performance of these analyzers in assessing precision and conducting method comparisons to determine the diagnostic accuracy and clinical efficacy of the Dymind automated system.

**Methods:**

We assessed precision and conducted a method comparison by analyzing complete blood count (CBC) and white blood cell (WBC) differential data from the Dymind DF55, DH76, and DH615. Results were compared with those from the Sysmex XN-3000 analyzer.

**Results:**

The Dymind-series analyzers demonstrated high between-run precision for all CBC and WBC differential parameters. Method comparison revealed a strong correlation (r = 0.80–0.99) between the Dymind and Sysmex analyzers for most CBC parameters, except for mean corpuscular hemoglobin concentration and basophil counts.

**Conclusions:**

The Dymind-series analyzer exhibited a strong analytical performance across all standard CBC and WBC differential count parameters, validating their precision and comparability with a reference system for routine hematological testing.

## Highlights

• Evaluated the performance of Dymind automated hematology analyzers (DF55, DH76, and DH615).
• Demonstrated high between-run precision for CBC and WBC differential parameters.
• Strong correlation (r = 0.80–0.99) with the Sysmex XN-3000 analyzer across most parameters.
• Validates Dymind analyzers for reliable routine hematological testing.

## Graphical abstract

**Figure:**
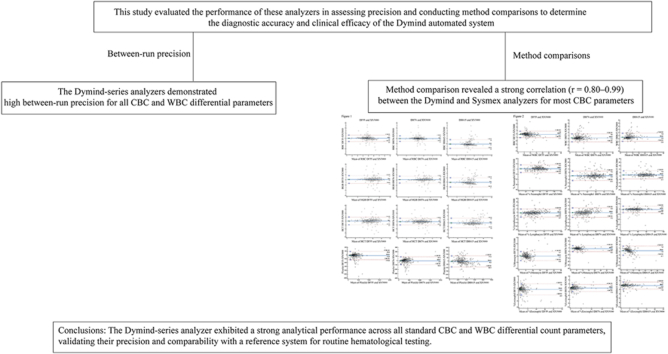

## 1 Introduction

Complete blood count (CBC), including white blood cell (WBC) differential analysis, is one of the most frequently requested tests in clinical diagnostics. Dymind automated hematology analyzers (Dymind, Shenzhen, China) include the five-part hematology analyzers DF55 and DH76, which can analyze 60 and 90 CBC/differential (CBC/DIFF) samples per hour, respectively. The six-part hematology analyzer, DH615, incorporates reticulocyte parameters and artificial intelligence (AI)-driven analysis technology. Current hematological laboratory guidelines recommend evaluating the performance of a new instrument before clinical use [[1], [2], [3], [4], [5]]. Although the precision and method comparison of the DH76 with the Sysmex XN-1000 have been reported [6], the performance of the new Dymind automated hematology analyzers in our population remains unclear. We aimed to evaluate the performance of the new Dymind automated hematology analyzers (DF55, DH76, and DH615) by assessing precision and comparing CBC parameters with those from Sysmex XN-3000.

## 2 Materials and methods

### 2.1 Patient samples

Residual ethylenediaminetetraacetic acid (EDTA)-anticoagulated blood samples from the Department of Pathology, Faculty of Medicine, Prince of Songkla University were processed within 6 h of collection [7]. The initial analysis of all specimens was performed using a Sysmex XN-3000 automated hematology analyzer to obtain CBC results. Selected samples were subsequently used to evaluate the analytical performance of the Dymind-series analyzers. This study was conducted in accordance with the Declaration of Helsinki and approved by the Ethics Committee of the Human Research Ethics Unit, Faculty of Medicine, Prince of Songkla University (Approval ID: REC.68-050-5-2).

### 2.2 Instrument

Three hematology analyzers—DF55, DH76, and DH615 (Shenzhen Dymind Biotechnology Co., Ltd., Guangming, Shenzhen, China)—were evaluated. The Dymind DF55 is a compact model (364 mm × 498 mm × 431 mm) with a throughput of 60 CBC/DIFF samples per hour. The Dymind DH76 is an enhanced throughput model that analyzes 90 samples per hour. The Dymind DH615 is an advanced model incorporating AI technology (665 mm × 870 mm × 820 mm). In our study, all analyzers used the electrical impedance method for red blood cell (RBC), WBC, and platelet counts. Hemoglobin (HGB) was measured using a cyanide-free colorimetric method. The DF55 and DH76 analyzers used semiconductor laser-based flow cytometry for WBC differentiation, whereas the DH615 used laser-scattering flow cytometry with fluorescence detection.

### 2.3 Precision assessment

Quality control procedures were performed using commercial control materials. DM-5D controls at three concentrations (low, normal, and high) were used for DF55 and DH76, whereas DM-6D controls at the same concentrations were used for DH615. These controls were used to monitor the system performance for CBC, WBC six-part differential, and nucleated red blood cell parameters.

According to the Clinical and Laboratory Standards Institute (CLSI) guideline EP5-A2 [4,8], between-run precision was measured by processing the manufacturer's quality control materials from three different lot numbers (low, normal, and high) twice daily for 20 consecutive days. The mean, standard deviation, and coefficient of variation (CV) were calculated for WBC, monocyte distribution width (MDW), RBC, HGB, hematocrit (HCT), mean corpuscular volume, mean corpuscular hemoglobin, mean corpuscular hemoglobin concentration (MCHC), red distribution width (RDW), platelet count, mean platelet volume (MPV), absolute neutrophils, lymphocytes, monocytes, eosinophils, and basophil counts.

### 2.4 Method comparison

CBC parameters obtained from the Dymind analyzers were compared with the results from the Sysmex XN-3000 (Sysmex, Kobe, Japan) using 483 samples for DF55 and DH76 and 371 samples for DH615. Outliers with abnormal scattergrams were excluded (58 for DF55/DH76 and 26 for DH615), resulting in 425 samples for DF55 and DH76 and 345 samples for DH615. The correlation coefficients, regression equation, difference, and percentage difference of the CBC parameter comparison were calculated using CLSI EP9-A2 [3].

### 2.5 Statistical analysis

Statistical analyses were performed using Microsoft Excel (Microsoft Corporation, Redmond, WA, USA) and the MedCalc software (free trial version) (MedCalc Software Inc., Mariakerke, Belgium). Data distribution was assessed using the Shapiro–Wilk test. For the assessment of method comparisons, Passing–Bablok linear regression analysis and Spearman correlations were used to determine the degree of correlation between the Sysmex XN-3000 and Dymin series. Bland–Altman difference plots were used to evaluate the absolute differences. The correlation coefficients and biases of the samples were determined. Statistical comparisons were considered significant at *p* < 0.05.

## 3 Results

### 3.1 Precision

The between-run precision results are provided in Table 1. Most RBC parameters measured using the Dymind analyzer demonstrated excellent precision, with CV below 2 % across all three levels of quality control. Exceptions were observed for the Dymind DF55 analyzer, where HGB and HCT at the low control level, as well as HCT at the high control level, showed slightly higher CVs ranging from 2.0 % to 2.3 %. For the Dymind DH76 analyzer, low-level controls for HGB, HCT, and RDW exhibited CVs of approximately 2 %. Dymind DH615 showed CVs between 2.0 % and 2.4 % for low-level controls of HGB, HCT, MCHC, and RDW, as well as for normal and high-level controls of HCT. For platelet counts, CVs remained below 5 % across all three analyzers, and the MPV was consistently <4 %. WBC counts demonstrated between-run precision with CVs below 2.4 %. The absolute counts of neutrophils and lymphocytes showed CVs between 1 % and 5 %, whereas those of monocytes, eosinophils, and basophils exhibited CVs ranging from 1 % to 6 %.

**Table 1:** Between-run precision of CBC and five-part differential (5-DIFF) count on Dymind DF55, DH76, and DH615 automated hematology analyzer.

Parameter	Low						Normal						High					
	DF55		DH76		DH615		DF55		DH76		DH615		DF55		DH76		DH615	
	Mean (SD)	%CV	Mean (SD)	%CV	Mean (SD)	%CV	Mean (SD)	%CV	Mean (SD)	%CV	Mean (SD)	%CV	Mean (SD)	%CV	Mean (SD)	%CV	Mean (SD)	%CV
RBC count ( × 10^12^/μL)	2.36 (0.04)	1.82	2.36 (0.04)	1.82	2.36 (0.04)	1.82	4.73 (0.06)	1.29	4.56 (0.05)	1.13	4.40 (0.04)	0.90	5.37 (0.08)	1.50	5.37 (0.08)	1.50	5.44 (0.05)	0.87
HGB (g/dL)	5.92 (0.12)	2.01	5.92 (0.12)	2.01	5.92 (0.12)	2.01	13.50 (0.19)	1.41	13.64 (0.14)	1.02	13.08 (0.08)	0.64	17.21 (0.72)	1.58	17.21 (0.72)	1.58	17.16 (0.11)	0.64
HCT (%)	18.82 (0.43)	2.28	18.82 (0.43)	2.28	18.82 (0.43)	2.28	41.35 (0.61)	1.47	40.19 (0.44)	1.11	40.30 (0.81)	2.01	40.30 (0.81)	2.01	51.09 (0.84)	1.65	52.57 (1.08)	2.06
MCV (fL)	79.85 (0.93)	1.17	77.75 (0.72)	0.93	79.63 (1.24)	1.56	87.45 (0.64)	0.73	88.17 (0.61)	0.69	91.35 (1.51)	1.65	95.14 (0.61)	0.64	96.13 (0.59)	0.61	96.39 (1.53)	1.59
MCH (pg)	25.11 (0.27)	1.08	25.67 (0.40)	1.55	26.13 (0.24)	0.94	28.54 (0.25)	0.87	29.93 (0.39)	1.30	29.72 (0.23)	0.76	32.03 (0.32)	0.99	32.65 (0.36)	1.11	31.50 (0.23)	0.72
MCHC (g/dL)	31.46 (0.53)	1.68	33.02 (0.54)	1.64	32.52 (0.79)	2.43	32.64 (0.35)	1.07	33.94 (0.37)	1.10	32.77 (0.41)	1.24	33.68 (0.38)	1.12	33.98 (0.33)	0.97	33.12 (0.43)	1.24
RDW	16.81 (0.29)	1.72	19.60 (0.40)	2.02	15.87 (0.35)	2.18	14.50 (0.16)	1.08	16.27 (0.20)	1.21	14.20 (0.22)	1.52	13.57 (0.18)	1.29	14.91 (0.21	1.42	13.51 (0.17)	1.26
Platelet count ( × 10^9^/μL)	71.47 (3.35)	4.70	54.69 (2.85)	5.22	79.67 (3.34)	4.19	270.88 (5.60)	2.06	252.42 (6.87)	2.73	300.47 (9.62)	3.20	502.61 (8.04)	1.61	489.16 (0.21)	2.81	573.25 (12.46)	2.17
MPV (fL)	8.74 (0.32)	3.70	9.40 (0.33)	3.52	7.46 (0.17)	2.23	8.60 (0.16)	1.91	9.06 (0.12)	1.38	7.38 (0.12)	1.69	8.87 (0.13)	1.50	9.12 (0.10)	1.15	7.19 (0.08)	1.14
WBC count ( × 10^9^/μL)	3.79 (0.09)	2.37	3.62 (0.08)	2.27	4.19 (0.07)	1.69	8.67 (0.21)	2.42	8.47 (0.16)	1.91	7.89 (0.10)	1.32	19.46 (0.39)	2.03	19.48 (0.33)	1.70	23.26 (0.23)	0.98
5-part differential count ( × 10^9^/μL)																		
Neutrophil	1.87 (0.06)	2.96	1.76 (0.07)	3.82	2.92 (0.07)	1.49	4.95 (0.15)	3.14	4.82 (0.15)	3.15	4.75 (0.08)	1.68	12.42 (0.32)	2.60	12.50 (0.28)	2.27	15.92 (0.19)	1.17
Lymphocyte	1.54 (0.05)	3.24	1.46 (0.06)	4.17	0.65 (0.03)	4.76	2.64 (0.08)	3.20	2.59 (0.07)	2.88	2.24 (0.05)	2.14	3.99 (0.11)	2.82	4.16 (0.14)	3.51	5.20 (0.07)	1.34
Monocyte	0.15 (0.01)	5.20	0.21 (0.01)	5.18	0.35 (0.02)	5.03	0.38 (0.02)	5.46	0.51 (0.03)	5.02	0.50 (0.03)	5.07	0.97 (0.05)	5.50	1.22 (0.06)	4.87	0.89 (0.04)	4.35
Eosinophil	0.22 (0.01)	6.29	0.16 (0.01)	5.51	0.27 (0.01)	5.32	0.71 (0.04)	6.30	0.53 (0.03)	5.41	0.39 (0.03)	6.47	2.08 (0.08)	4.00	1.52 (0.08)	5.00	1.26 (0.04)	3.42
Basophil	0.15 (0.01)	6.22	2.15 (0.09)	4.17	3.34 (0.07)	2.07	0.22 (0.01)	5.99	6.00 (0.12)	1.96	5.59 (0.08)	1.44	0.24 (0.01)	6.20	15.80 (0.26)	1.66	16.89 (0.19)	1.14

Note: SD, standard deviation; RBC, red blood cell; WBC, white blood cell; MCV, mean corpuscular volume; MCH, mean corpuscular hemoglobin; MCHC, mean corpuscular hemoglobin concentration; MPV, mean platelet volume; red distribution width; CV, coefficient of variation; RDW, red distribution width; HGB, Hemoglobin; HCT, hematocrit; CV, coefficient of variation.

### 3.2 Method comparison

**Fig. 1:**
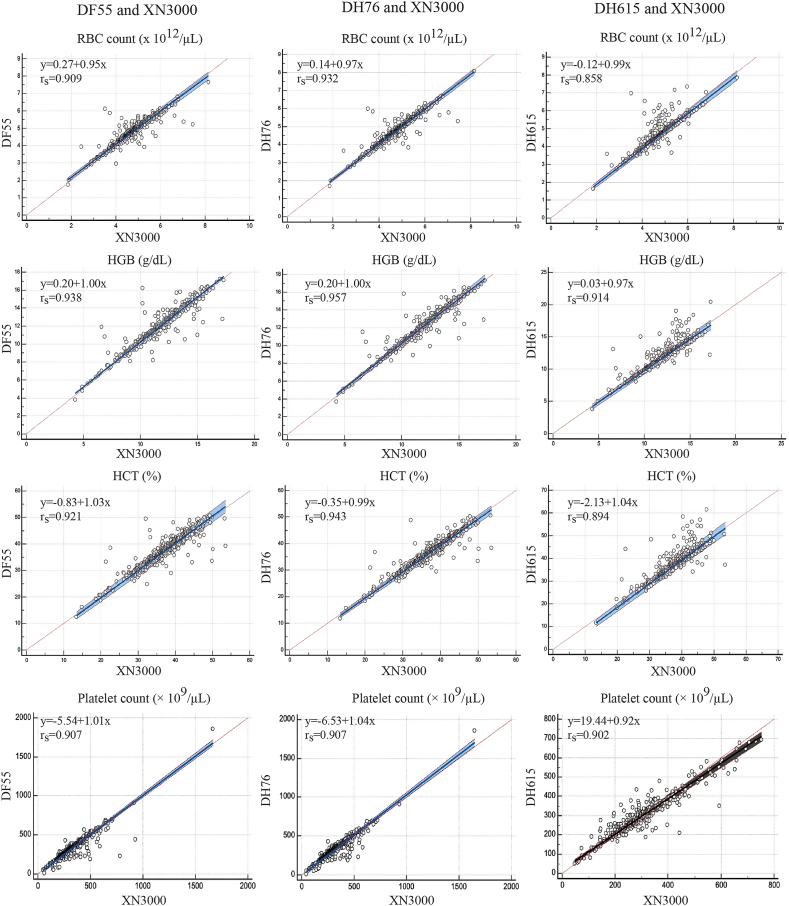
Passing–Bablok regression and Spearman correlation analysis (rs) comparing measurements obtained from the Dymind DF55, DH76, and DH615 hematology analyzers with those from the Sysmex XN-3000 analyzer for red blood cell (RBC) parameters: RBC count, hemoglobin (HGB), hematocrit (HCT), and platelet count. RBC, red blood cell; HGB, hemoglobin; HCT, hematocrit.

**Fig. 2:**
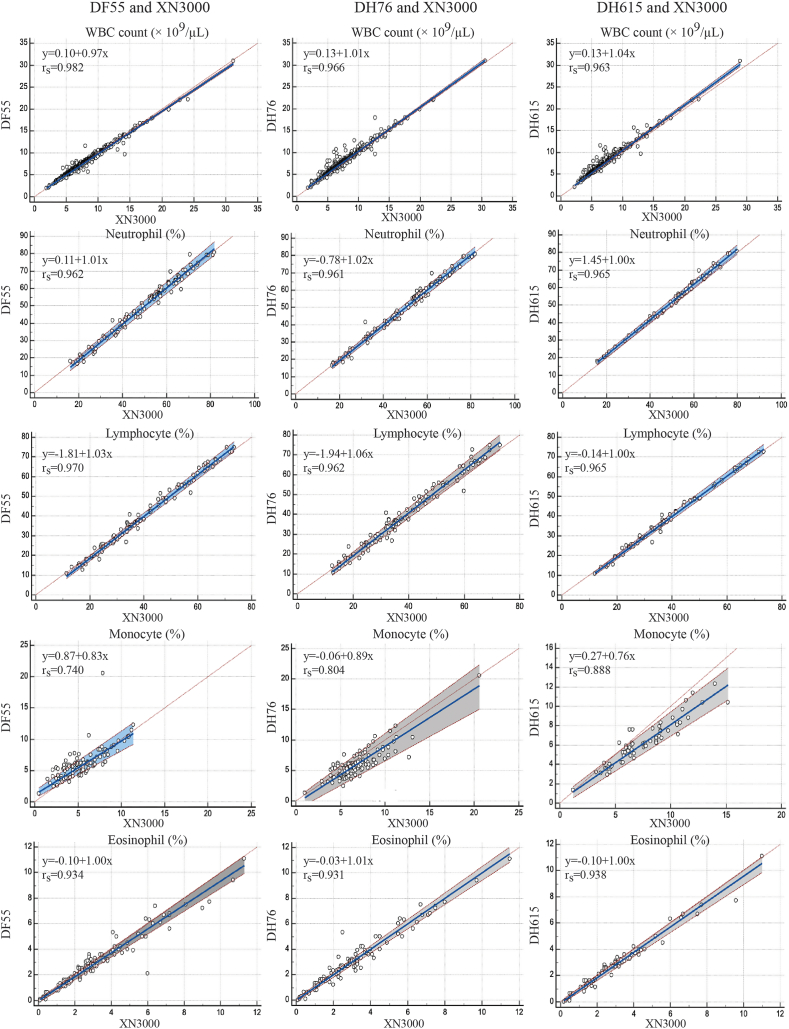
Passing–Bablok regression and Spearman correlation analysis (rs) comparing measurements obtained from the Dymind DF55, DH76, and DH615 hematology analyzers with those from the Sysmex XN-3000 analyzer for for white blood cell (WBC) count; % neutrophil, % lymphocyte, % monocyte, and % eosinophil in blood samples.

**Fig. 3:**
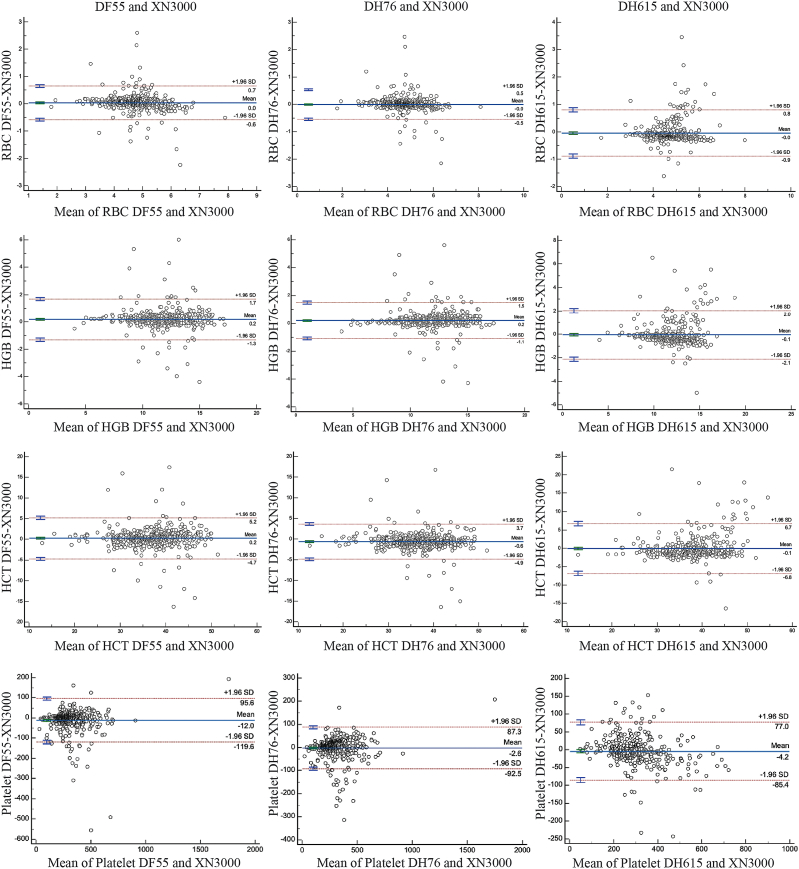
Bland−Altman plots from the comparison among the Dymind DF55, DH76, and DH615 hematology analyzer and Sysmex XN3000 analyzer measurements for RBC parameter; RBC count, HGB, HCT, and platelet count in blood samples. Bland−Altman difference plots are shown with bias (solid blue line) and ±1.96 SD (dashed red lines). RBC, red blood cell; HGB, Hemoglobin; HCT, hematocrit; SD, standard deviation.

**Fig. 4:**
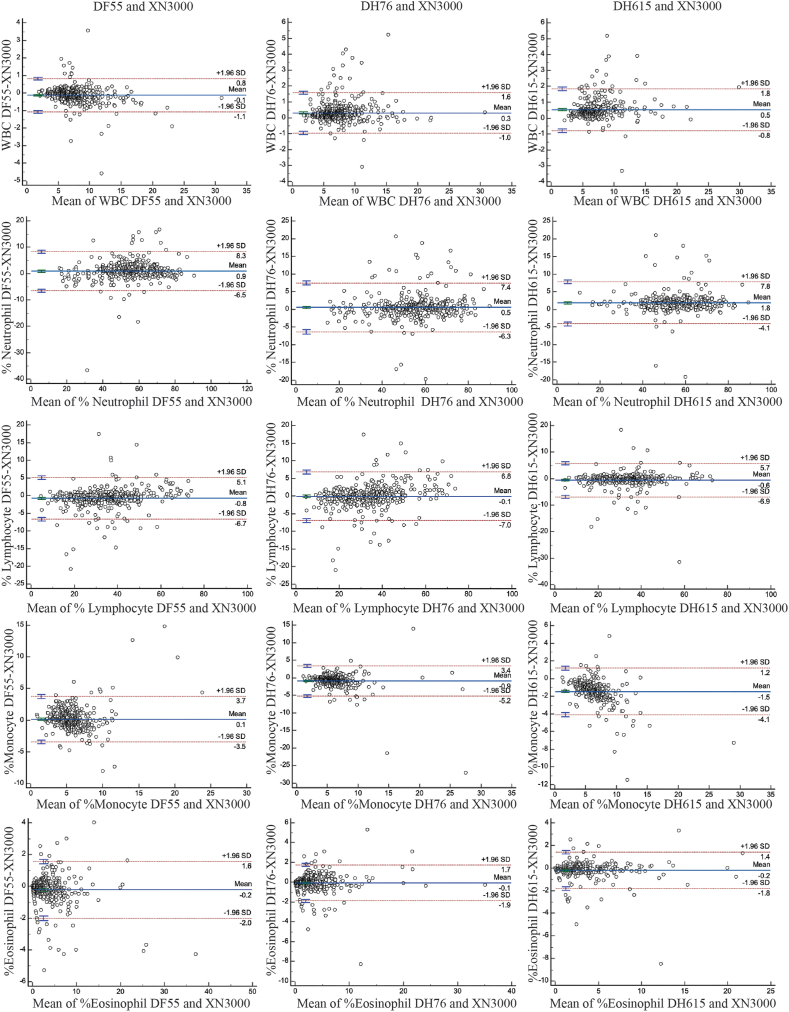
Bland−Altman plots from the comparison among the Dymind DF55, DH76, and DH615 hematology analyzer and Sysmex XN3000 analyzer measurements for white blood cell (WBC) count; % neutrophil, % lymphocyte, % monocyte, and % eosinophil in blood samples. Bland−Altman difference plots are shown with bias (solid blue line) and ±1.96 SD (dashed red lines). SD, standard deviation.

The concentration range of each parameter used for method comparison is presented in Table 2. A comparative analysis using Passing–Bablok regression of the Dymind analyzers against the Sysmex XN-3000 demonstrated strong correlations across most CBC parameters (Fig. 1, Fig. 2, and Table 3). For RBC parameters, the correlation coefficients exceeded 0.90 for all metrics except MCHC, which showed a moderate correlation (rs > 0.70). Platelet count and MPV exhibited a strong correlation (rs > 0.90) via Passing–Bablok regression. The WBC count, percentage of neutrophils, and percentage of lymphocytes correlated well between the Dymind series and Sysmex XN-3000, with r > 0.96. Monocyte percentage correlations varied by model, with rs values > 0.89 for DH615, >0.80 for DH76, and >0.74 for DF55. Eosinophil percentages showed high agreement across all analyzers, with correlation coefficients exceeding 0.93 (Fig. 1, Fig. 2, and Table 3). Bland–Altman analyses showed a statistically significant positive bias for RBC count, HGB, HCT, and the percentages of neutrophils and monocytes, and a statistically significant negative bias for platelet count, WBC count, and the percentages of lymphocytes and eosinophils in the Dymind DF55. For the Dymind DH76 analyzer, Bland–Altman bias analysis revealed a statistically significant positive bias for HGB, WBC count, and the percentage of neutrophils, and a statistically significant negative bias for RBC count, HCT, platelet count, and the percentages of lymphocytes, monocytes, and eosinophils. However, this analysis exhibited a statistically significant positive bias for WBC count and the percentage of neutrophils, and a statistically significant negative bias for RBC count, HGB, HCT, platelet count, the percentages of lymphocytes and monocytes, and eosinophils in the Dymind DH615 analyzer (Fig. 3, Fig. 4).

**Table 2:** Concentration ranges of hematological parameters in the comparison samples.

Parameter	DF55 (N = 483)	DH76 (N = 483)	DH615 (N = 371)	XN3000 (N = 483)
RBC count ( × 10^12^/μL)	1.74–7.64	1.70–8.06	1.63–7.85	1.86–8.16
HGB (g/dL)	3.80–17.20	3.70–17.30	3.80–20.40	4.30–17.30
HCT (%)	12.40–50.70	11.80–50.50	11.60–61.50	13.40–53.50
MCV (fL)	49.60–119.4	47.60–119.1	52.80–119.0	51.20–115.50
MCH (pg)	15.10–38.70	14.60–39.60	15.00–40.00	14.60–41.00
MCHC (g/dL)	26.10–34.70	26.20–34.90	27.10–34.60	24.50–36.60
RDW	12.80–31.60	12.40–33.60	12.40–26.30	11.00–31.50
Platelet count ( × 10^9^/μL)	50.00–1666	46.00–1651	48.00–752.0	50.00–1857
WBC count ( × 10^9^/μL)	1.90–31.18	1.84–30.56	2.22–28.95	1.96–30.89
5-part differential count (%)				
Neutrophil	15.0–91.20	14.90–89.50	8.30–88.50	13.00–90.36
Lymphocyte	4.80–73.50	5.00–72.90	5.20–73.50	3.00–74.90
Monocyte	0.50–21.70	1.00–41.10	1.20–32.70	1.00–26.00
Eosinophil	0.00–39.30	0.10–35.30	0.00–21.40	0.00–35.00
Basophil	0.10–3.30	0.00–3.00	0.00–2.30	0.00–3.00

Note: RBC, red blood cell; WBC, white blood cell; MCV, mean corpuscular volume; MCH, mean corpuscular hemoglobin; MCHC, mean corpuscular hemoglobin concentration; red distribution width; RDW, red distribution width; HGB, Hemoglobin; HCT, hematocrit.

**Table 3:** Passing–Bablok regression analysis for Dymind DF55, DH76, and DH615 and Sysmex XN3000 method comparison.

Parameter	DF55 and XN3000				DH76 and XN3000				DH615 and XN3000			
	N	*r*_*s*_	Slope (95 % CI)	Intercept A (95 % CI)	N	*r*_*s*_	Slope (95 % CI)	Intercept A (95 % CI)	N	*r*_*s*_	Slope (95 % CI)	Intercept A (95 % CI)
RBC count ( × 10^12^/μL)	483	0.909	0.95 (0.93, 0.98)	0.27 (0.18, 0.35)	483	0.932	0.97 (0.96, 0.98)	0.14 (0.07, 0.19)	371	0.858	0.99 (0.97, 1.01)	−0.12 (−0.21, −0.02)
HGB (g/dL)	483	0.938	1.00 (1.00, 1.00)	0.20 (0.20, 0.20))	483	0.957	1.00 (1.00, 1.03)	0.20 (−0.09, 0.20)	371	0.914	0.97 (0.95, 1.00)	0.03 (−0.30, 0.28)
HCT (%)	483	0.921	1.03 (1.00, 1.05)	−0.83 (−1.71, 0.20)	483	0.943	0.99 (0.97, 1.01)	−0.35 (−0.85, 0.40)	371	0.894	1.04 (1.01, 1.07)	−2.13 (−3.52, −1.25)
MCV (fL)	483	0.988	1.00 (0.99, 1.02)	−0.30 (−1.50, 0.47)	483	0.989	1.04 (1.03, 1.06)	−4.61 (−5.68, −3.53)	371	0.988	1.02 (1.01, 1.04)	−1.30 (−2.47, −0.03)
MCH (pg)	483	0.992	0.91 (0.90, 0.92)	2.43 (2.17, 2.68)	483	0.993	1.01 (1.00, 1.02)	0.25 (−0.1, 0.40)	371	0.992	0.98 (0.97, 0.99)	0.68 (0.35, 0.98)
MCHC (g/dL)	483	0.707	0.60 (0.55, 0.64)	13.00 (11.69, 14.55)	483	0.770	0.65 (0.61, 1.08)	12.10 (10.76, 13.48)	371	0.805	0.59 (0.55, 0.63)	13.00 (11.93, 14.18)
RDW	483	0.975	0.73 0.71, 0.75)	4.92 (4.68, 5.20)	483	0.978	0.85 (0.83, 0.88)	3.23 (2.93, 3.53)	371	0.972	0.66 (0.64, 0.68)	5.37 (5.13, 5.67)
Platelet count ( × 10^9^/μL)	483	0.907	1.01 (0.99, 1.03)	−5.54 (−11.32,−0.06)	483	0.907	1.04 (1.01, 1.06)	−6.53 (−13.34, 0.01)	371	0.902	0.92 (0.89, 0.94)	19.44 (12.46, 26.04)
MPV (fL)	483	0.880	1.05 (1.00, 1.11)	0.86 (0.38, 1.30)	425	0.905	0.97 (0.92, 1.00)	1.16 (0.90, 1.67)	371	0.905	1.00 (1.00, 1.00)	0.80 (0.80, 0.80)
WBC count ( × 10^9^/μL)	483	0.982	0.97 (0.96, 0.98)	0.10 (0.04, 0.17)	483	0.966	1.01 (1.00, 1.03)	0.13 (0.06, 0.20)	371	0.963	1.04 (1.03, 0.93)	0.13 (0.03, 0.23)
5-part differential count (%)												
Neutrophil	425	0.962	1.01 (1.00, 1.03)	0.11 (−0.85, 1.01)	425	0.961	1.02 (1.01, 1.03)	−0.78 (−1.55, 0.01)	345	0.965	1.00 (0.99, 1.02)	1.45 (0.74, 2.11)
Lymphocyte	425	0.970	1.03 (1.02, 1.05)	−1.81 (−2.29, −1.38)	425	0.962	1.06 (1.04, 1.08)	−1.94 (−2.51, −1.35)	345	0.965	1.00 (0.99, 1.02)	−0.41 (−0.92, −0.06)
Monocyte	425	0.740	0.83 (0.78, 0.90)	0.87 (0.52, 1.19)	425	0.804	0.89 (0.85, 0.94)	−0.06 (−0.34, 0.20)	345	0.888	0.76 (0.73, 0.79)	0.27 (0.08, 0.44)
Eosinophil	425	0.934	1.00 (−1.27, 1.27)	−0.10 (−0.10, −0.07)	425	0.931	1.01 (1.00, 1.04)	−0.03 (−0.13, 0.00)	345	0.938	1.00 (1.00, 1.02)	−0.10 (−0.19, −0.10)
Basophil	425	0.451	0.71 (0.63, 0.80)	−0.01 (−0.08, 0.05)	425	0.179	0.20 (0.14, 0.25)	0.14 (0.10, 0.16)	345	0.161	2.00 (2.00, 3.00)	−0.10 (−0.40, −0.10)

Note: rs, Spearman correlations correlation coefficients; SD, standard deviation; RBC, red blood cell; WBC, white blood cell; MCV, mean corpuscular volume; MCH, mean corpuscular hemoglobin; MCHC, mean corpuscular hemoglobin concentration; MPV, mean platelet volume; CI, confidence interval; RDW, red distribution width; HGB, Hemoglobin; HCT, hematocrit.

## 4 Discussion

CBC is one of the most frequently ordered laboratory tests and is crucial in the initial diagnosis and ongoing monitoring of various clinical conditions. Recent advancements in automated hematology analyzers have significantly enhanced the speed, accuracy, and range of available hematological parameters. This study evaluated the analytical performance of three models from the Dymind series—DF55, DH76, and DH615—and demonstrated consistently strong performance across key metrics.

The between-run precision for all CBC and WBC differential parameters was excellent and within acceptable limits provided by the manufacturer. These results are comparable to those obtained from analyzers based on similar technological principles, such as the Mindray BC-5000 [9] and Sysmex XP-300 [10]. Dymind DH76 showed improved precision for platelet count and MPV within the normal range compared with previous studies [6].

Method comparison analyses demonstrated good agreement between the Dymind-series analyzers and the Sysmex XN-3000. Most CBC parameters, including RBC count, HGB, HCT, and platelet count, showed a strong correlation (r > 0.90), except for MCHC, which exhibited lower correlation coefficients of 0.707 for DF55 and 0.770 for DH76. Similar discrepancies for MCHC have been reported in comparisons with other analyzers, such as Abbott Alinity HQ and Sysmex XN‐9000 (r = 0.683) [11] and Roche Cobas M511 vs. Sysmex XN (r = 0.562) [12]. For WBC differential parameters, the Dymind analyzer demonstrated a strong correlation with the Sysmex XN-3000 for neutrophils, lymphocytes, and eosinophils (r > 0.93). The correlation for monocytes was comparatively lower at 0.740 for DF55, 0.804 for DH76, and 0.888 for DH615. These findings are consistent with previous reports on other hematology systems, such as the Abbott Alinity HQ vs. Sysmex XN‐9000 (r = 0.849) [11], and comparisons between DxH 800 and Sapphire (r = 0.774) and DxH 800 and LH 780 (r = 0.791) [13]. The correlation coefficients for basophils were low across all analyzers, consistent with observations from other published studies [13,14]. In this study, Bland–Altman analysis revealed systematic biases in several hematological parameters measured by the Dymind analyzers. Specifically, the DH76 and DH615 showed positive bias in WBC and neutrophil counts, while negative bias was observed in RBC, HCT, and platelet counts. Notably, all three models demonstrated a negative bias in platelet counts. This observation is consistent with previous reports highlighting discrepancies in platelet measurements among automated hematology analyzers, particularly in samples with low platelet counts [15,16]. The negative bias may reflect differences in detection principles, with the impedance-based system of the Dymind series compared to the optical-based methods of the Sysmex XN-3000. Although the observed biases were statistically significant, most fell within acceptable clinical limits, suggesting that the Dymind analyzers remain reliable for routine use. However, caution is warranted when interpreting results in patients with thrombocytopenia. Similarly, the positive bias observed in WBC and neutrophil counts in some models may affect the monitoring of infectious or inflammatory conditions.

To our knowledge, this is the first study to comprehensively evaluate the performance characteristics of the new Dymind DF55, DH76, and DH615 hematology analyzers. Our findings confirmed that all three models provided reliable and precise measurements of the standard CBC and WBC differential parameters, supporting the clinical utility of the Dymind series as a viable option for routine hematological analysis. Despite the strong analytical performance observed across all three Dymind hematology analyzers, some limitations should be acknowledged. First, this study was conducted in a single-center laboratory setting using residual samples, which may not fully reflect the performance of the analyzers across diverse clinical environments and patient populations. Multi-center evaluations involving a broader range of sample types—including pediatric, geriatric, and pathological specimens—would enhance generalizability of the findings. Furthermore, comparative clinical data on certain critical subgroups were not fully explored in this cohort. In particular, analyses of blood samples with low platelet counts should be expanded, as this range is especially important due to its association with higher coefficients of variation. Second, while our method comparison focused on standard CBC and WBC differential parameters, the potential utility of advanced features, particularly the artificial intelligence (AI)-driven analysis and reticulocyte parameters available in the DH615 model, was not fully explored. These parameters may offer diagnostic advantages, especially in complex hematologic conditions, and merit further targeted evaluation. Third, we excluded samples with abnormal scattergrams during method comparison, which may limit assessment under atypical or flagged conditions—circumstances that often pose the greatest diagnostic challenges in clinical practice. Future studies should aim to include flagged or atypical cases to better assess the robustness and interpretive accuracy of the analyzers under real-world conditions. Future work should also assess user experience, workflow efficiency, and integration within laboratory information systems, particularly in high-throughput settings. Incorporating cost-effectiveness analysis and turnaround time comparisons would provide a more comprehensive understanding of the analyzers’ utility in clinical laboratories.

## CRediT authorship contribution statement

**Tipparat Penglong:** Writing – review & editing, Resources, Methodology, Investigation, Formal analysis, Data curation. **Wanicha Tepakhan:** Resources, Methodology, Investigation, Formal analysis. **Nasra Tehyoh:** Methodology, Investigation. **Yanisa Na Songkhla:** Methodology, Investigation. **Chakkrit Songnak:** Methodology, Investigation. **Kanitta Srinoun:** Writing – original draft, Methodology, Investigation, Funding acquisition, Formal analysis, Data curation, Conceptualization.

## Disclosure statement

The authors declare no competing interests.

## Redundant publication

No substantial overlap with previous papers.

## Ethics approval statement

Ethics Committee of the Human Research Ethics Unit (HREU), Faculty of Medicine, Prince of Songkla University (Approval ID: REC.68-050-5-2).

## Data availability statement

Data is available on request due to privacy/ethical restrictions.

## Funding statement

Faculty of Medical Technology, Prince of Songkla University.

## Declaration of competing interest

Authors have no conflict of interest.

## Data Availability

Data will be made available on request.
